# Exploring the Neuroprotective Effects of Fingolimod in Colchicine‐Induced Sporadic Dementia in Rats

**DOI:** 10.1002/alz70858_100123

**Published:** 2025-12-25

**Authors:** Kajal Kajal, Smriti Thakur, Rahul Renukadas Deshmukh

**Affiliations:** ^1^ Central University of Punjab, Bathinda, Punjab, India; ^2^ Maharaja Ranjit Singh Punjab Technical University Dabwali Road, Bathinda, Punjab, India; ^3^ Department of Pharmacology, Central University of Punjab, Bathinda, Bathinda, Punjab, India

## Abstract

Alzheimer's disease (AD) is an age related neurodegenerative disease which is mainly characterized by progressive cognitive decline, synaptic loss, extracellular amyloid‐beta (Aβ) deposits and intracellular accumulation of neurofibrillary tangles of tau protein. Multiple etiological factors have been linked with AD pathophysiology, oxidative stress, neuroinflammation, neurotransmitter deficit and foremost are the deregulation of lipid metabolism, consistently involve in AD pathology and cognitive deficit. In the present study we have explored the neuroprotective potential of Fingolimod (FTY720), against intracerebroventricular colchicine (ICV‐COL) induced experimental sporadic dementia in rats. COL was administered bilaterally at a dose of (15μg/rat) on the first day. Spatial and non‐spatial memory was evaluated by using Morris water maze and object recognition test. Locomotor activity was evaluated by using open field test. Fingolimod (0.25 and 0.5 mg)/kg, p.o.) was administered on alternate days from 8th day after ICV ‐infusion in rats. The parameters of oxidative stress were accessed by measuring the malondialdehyde, reduced glutathione and catalase levels in the hippocampus and cortex region. ICV‐COL infusion in rats produced cognitive deficit and caused significant elevation in markers and oxidative stress and degenerative changes in hippocampus and cortical brain region. On the contrary, Fingolimod treatment attenuate COL‐induced cognitive decline, reduced oxidative burden and able to preserve the neuronal architecture and prevent neuronal loss in hippocampus and cortical brain region of the rats. The neurodegeneration in hippocampus and cortex regions was observed through histopathological examination. The outcome of the present study clearly indicates that the neuroprotective potential of fingolimod and suggesting its therapeutic potential in cognitive disorders.

